# Supplementary material for: Rescue of Mycobacterium bovis DNA Obtained from Cultured Samples during Official Surveillance of Animal TB: Key Steps for Robust Whole Genome Sequence Data Generation
Source: Int J Mol Sci. 2024 Mar 30;25(7):3869. doi: 10.3390/ijms25073869 (PMC11011339; doi:10.3390/ijms25073869)
Supplement: Supplementary file 1 [file ijms-25-03869-s001.zip › ijms-2937931-Supplementary Figures.pdf]

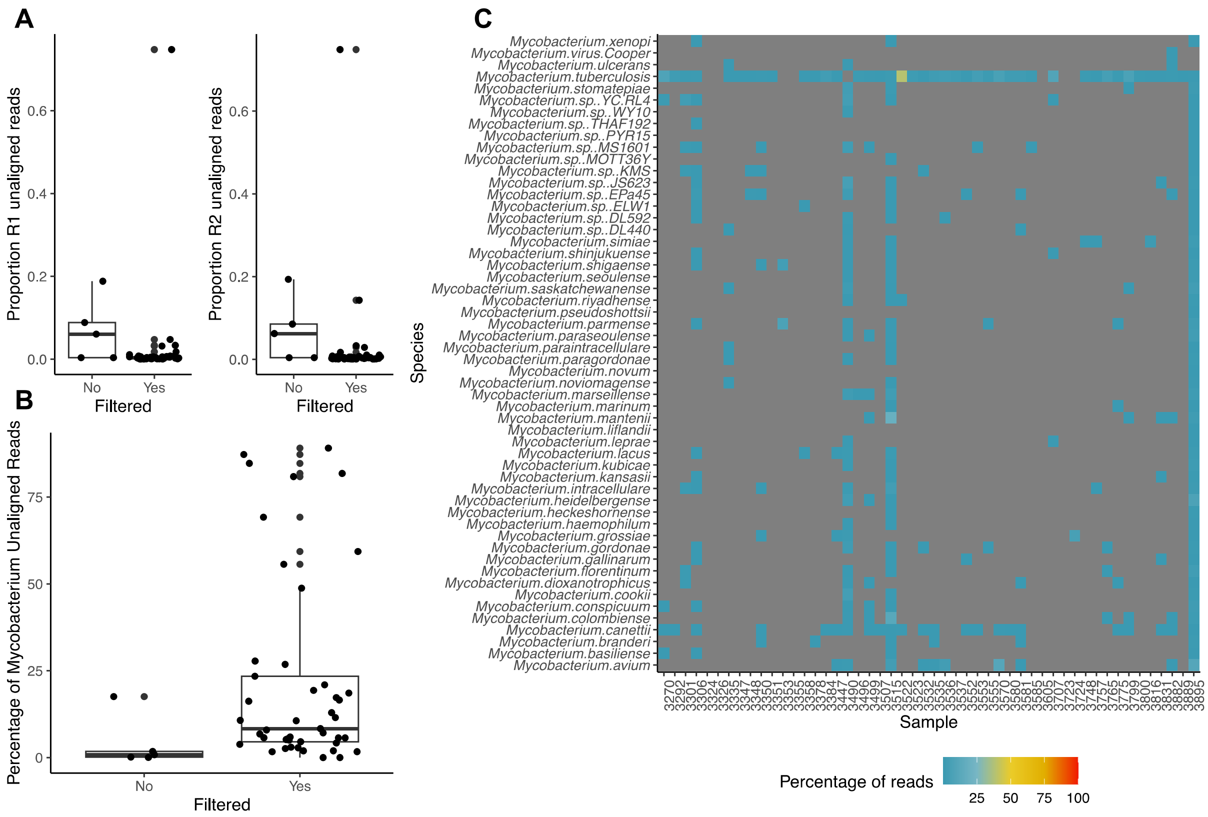

Figure S1. Contamination with non-tuberculous mycobacteria. (**A**) The distribution of the proportion of unaligned reads in both directions (R1 and R2) for Batch 3 samples that either were filtered to keep only *Mycobacterium* genus reads or not. (**B**) Percentage of those unaligned reads that once reclassified by Kraken were again classified as *Mycobacterium* reads. (**C**) Percentage of those *Mycobacterium* genus unaligned reads that were assigned to a given species. Grey means that no unaligned reads of that sample were assigned to that species.

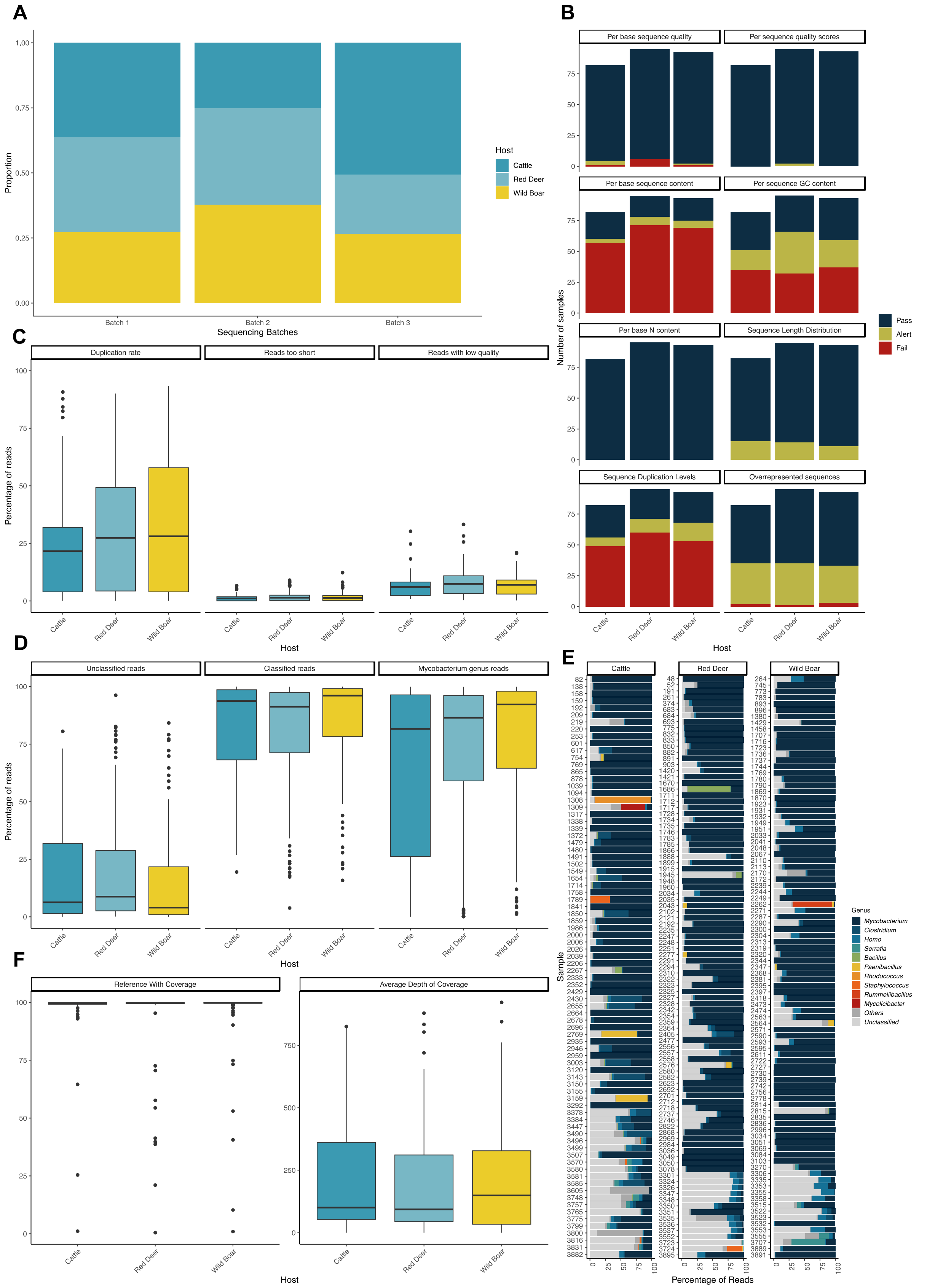

Figure S2. Sequencing metrics by the origin of the sample. (**A**) Distribution of host species in each sequencing batch. (**B**) Comparison of the sequencing reads quality parameters assessed by FastQC [21], divided by the origin of the

sample (cattle, red deer, or wild boar). FastQC classified the samples into three qualitative categories: Pass, Alert, and Fail. (**C**) Box plots of the distribution of the parameters analyzed by FastP [30] in the samples from the same dataset of panel A. The determined values of duplication rate, reads too short, and reads with low quality are percentages of the total of reads. (**D**) Box plots of the distribution of the Kraken2 [31] determined percentages of unclassified, classified, and mycobacterial reads in each sample. The samples were grouped by dataset, as in panel A. (**E**) Percentage of reads per sample classified in each of the genera indicated on the right. The "Unclassified" category comprises all the reads Kraken2 [31] was not able to classify and the "Other" category comprises the total of reads classified to other genera or not classified at the genus level. Represented are the ten most abundant genera in the full sample set. Sample origins are indicated on top. (**F**) Box plot of the distribution of the vSNP determined parameters "Average Depth of Coverage" and "Reference With Coverage".

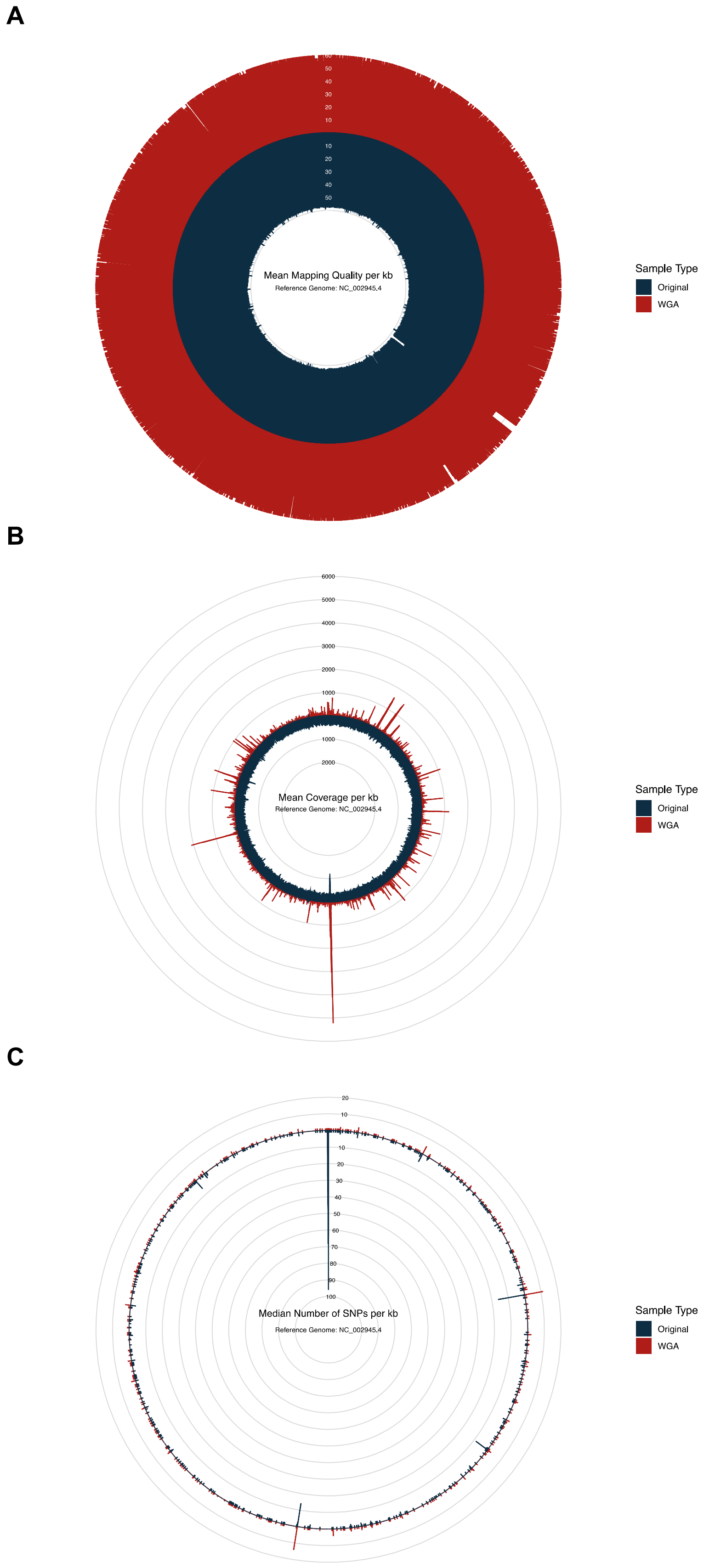

Figure S3. Comparison of mapping statistics between samples subjected or not to WGA. Twenty-five sequenced samples subjected to WGA and 26 randomly selected samples not subjected to WGA were analyzed. The circular

plots show how mapping quality (**A**), coverage (**B**) and SNP density (**C**) per kb change across the genome (on the circular axis). The data is color coded accordingly to the sample type (WGA or original).
